# Supplementary figures and images for: Quantifying transmission dynamics of acute hepatitis C virus infections in a heterogeneous population using sequence data
Source: PLoS Pathog. 2021 Sep 14;17(9):e1009916. doi: 10.1371/journal.ppat.1009916 (PMC8462723; doi:10.1371/journal.ppat.1009916)

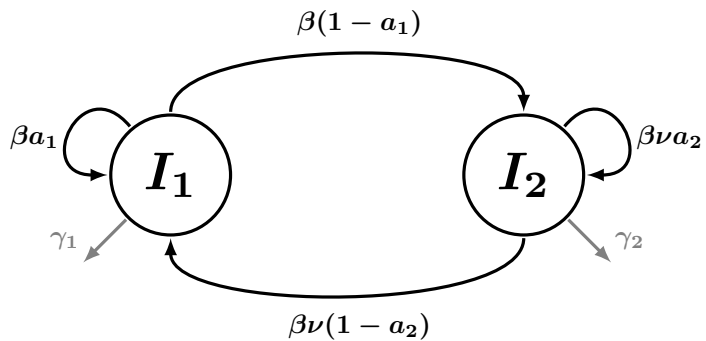

Supplement: S1 Fig — (PDF) [file ppat.1009916.s001.pdf]

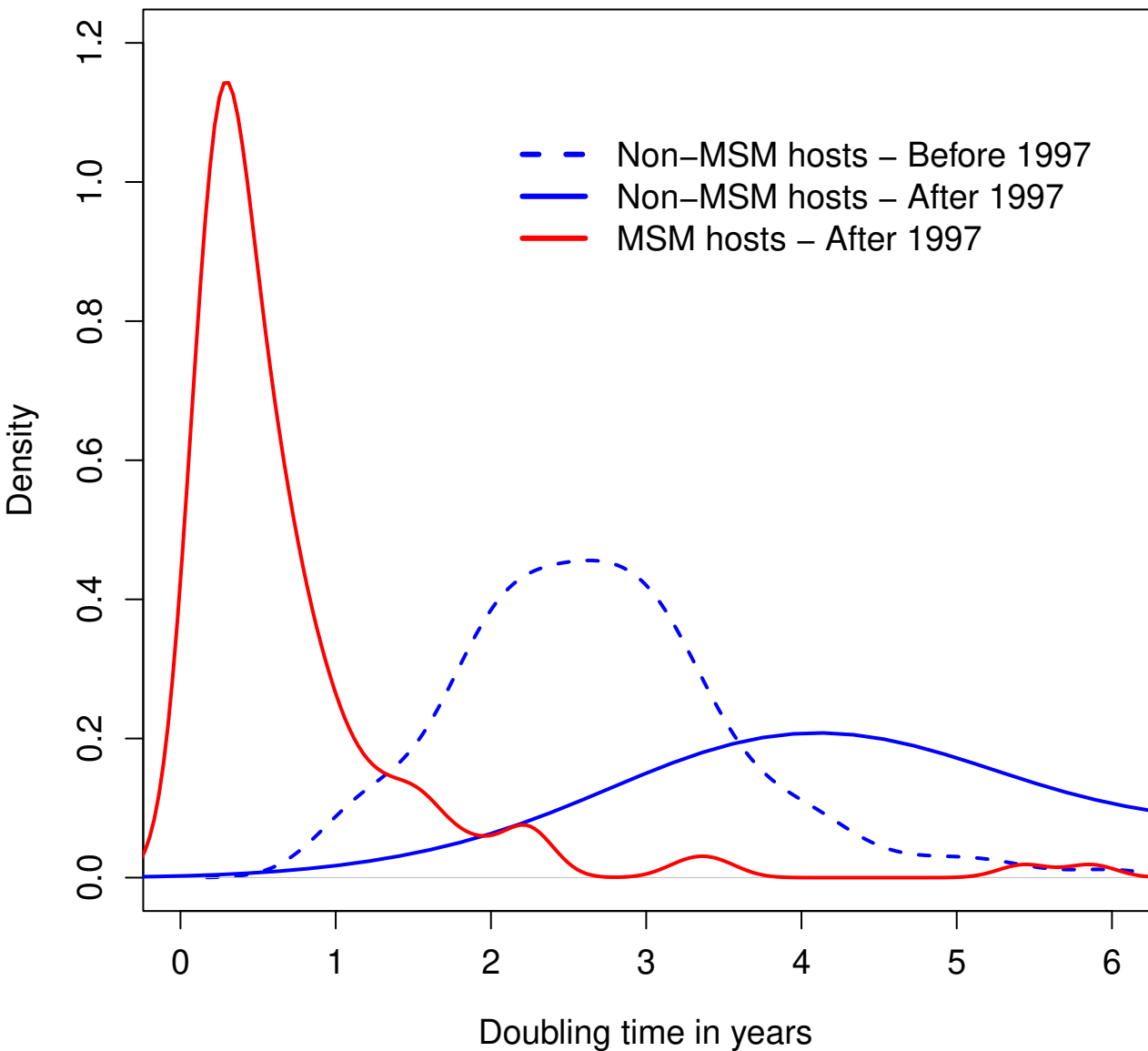

Supplement: S2 Fig — The density of the doubling time for the non-MSM hosts before 1997 (tD(2),t1) is in blue dashed line, and after 1997 (tD(1),t2) in blue solid line. The density of the doubling time for the MSM hosts (tD(2),t3) is in red. (PDF) [file ppat.1009916.s002.pdf]

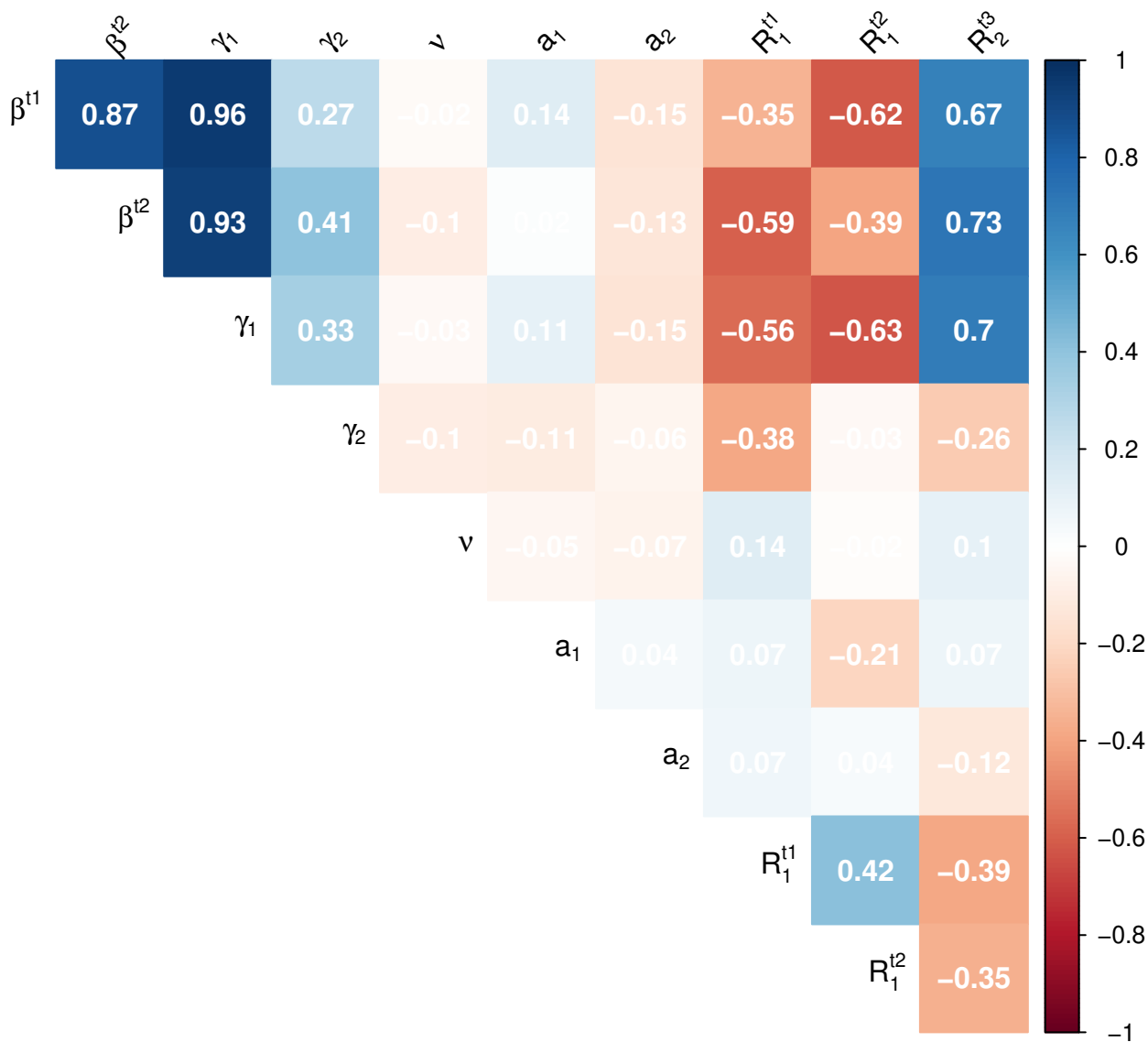

Supplement: S3 Fig — The intensity of the colour is proportional to the correlation coefficients. (PDF) [file ppat.1009916.s003.pdf]

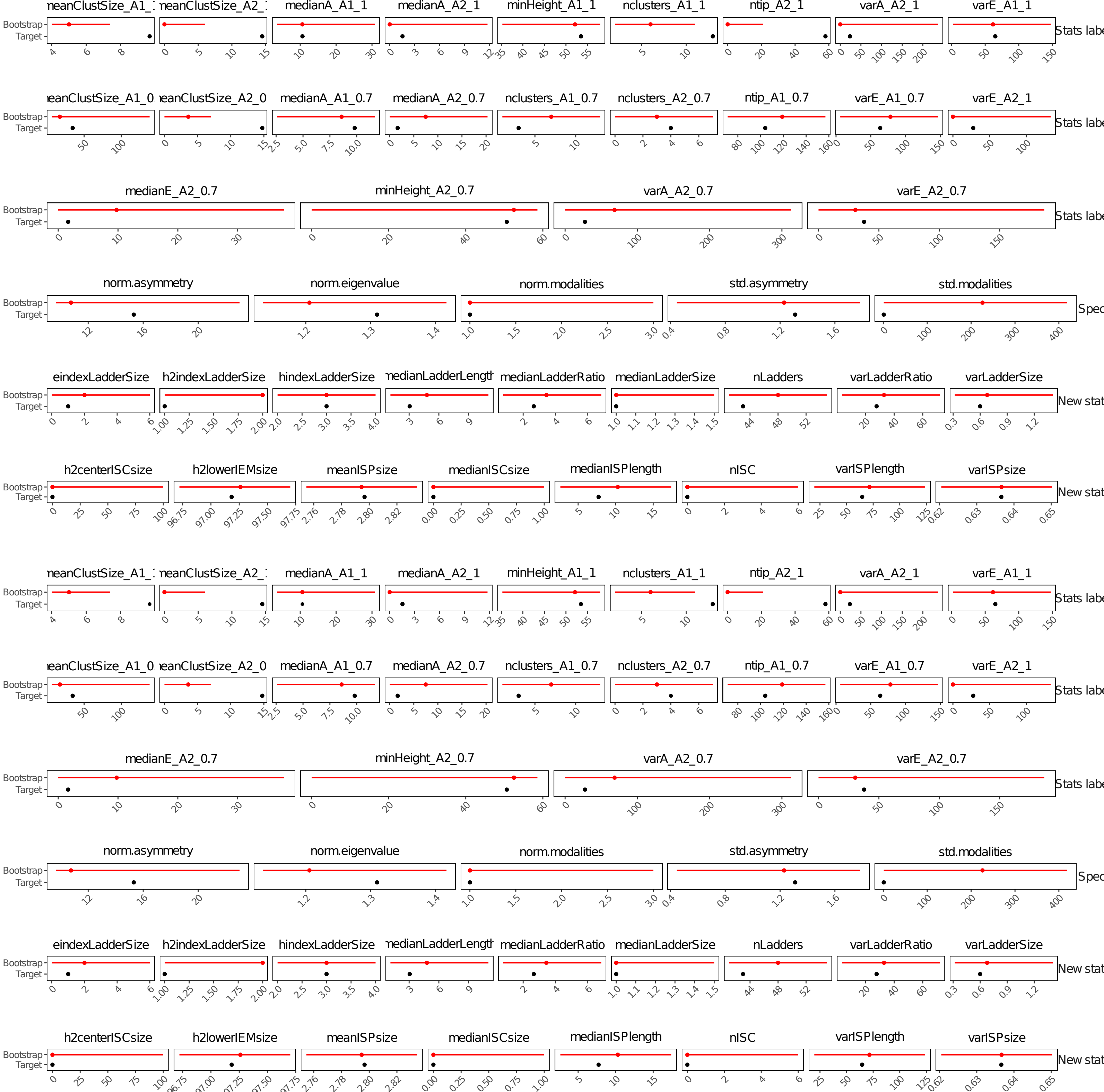

Supplement: S4 Fig — The dots represent the median and the horizontal lines represent the 95% HPD. Red distributions correspond to the summary statistics computed from the 10,000 phylogenies simulated from the posterior distribution. Black dots represent the values of selected summary statistics computed from the target phylogeny. Summary statistics are represented by group. (PDF) [file ppat.1009916.s004.pdf]

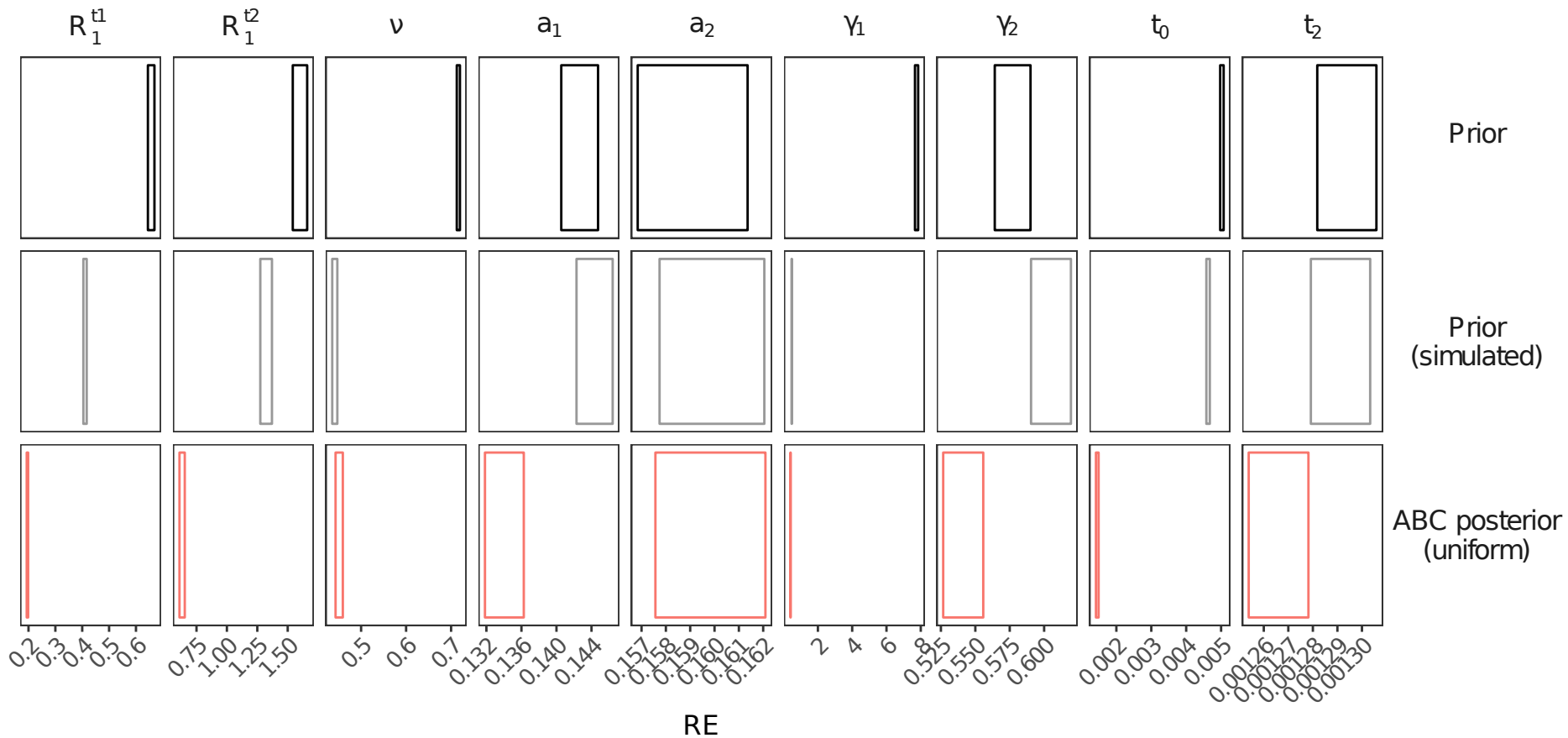

Supplement: S5 Fig — Each column corresponds to one of the inferred parameters. The first line shows the prior distribution. The second line shows the distribution of values for which a phylogeny could be simulated. The third line shows the inference after the ABC. For the rejection step of the ABC, the tolerance level was set to Pδ = 0.05. The rectangles show the mean relative errors and their standard errors computed for 100 target sets with known values (see the Material and methods). (PDF) [file ppat.1009916.s005.pdf]

Distribution/ABC Tolerance

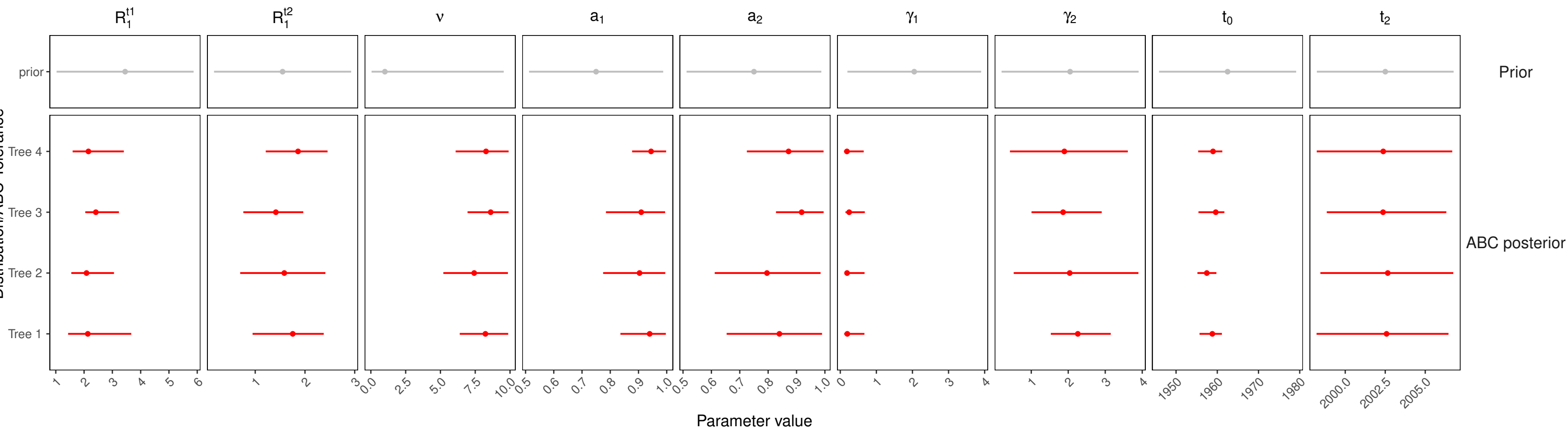

Supplement: S6 Fig — The first line represents the prior (in grey), the last line the full target tree (in red), and all the intermediate lines phylogenies where half of the MSM hosts’ sequences were removed at random. (PDF) [file ppat.1009916.s006.pdf]

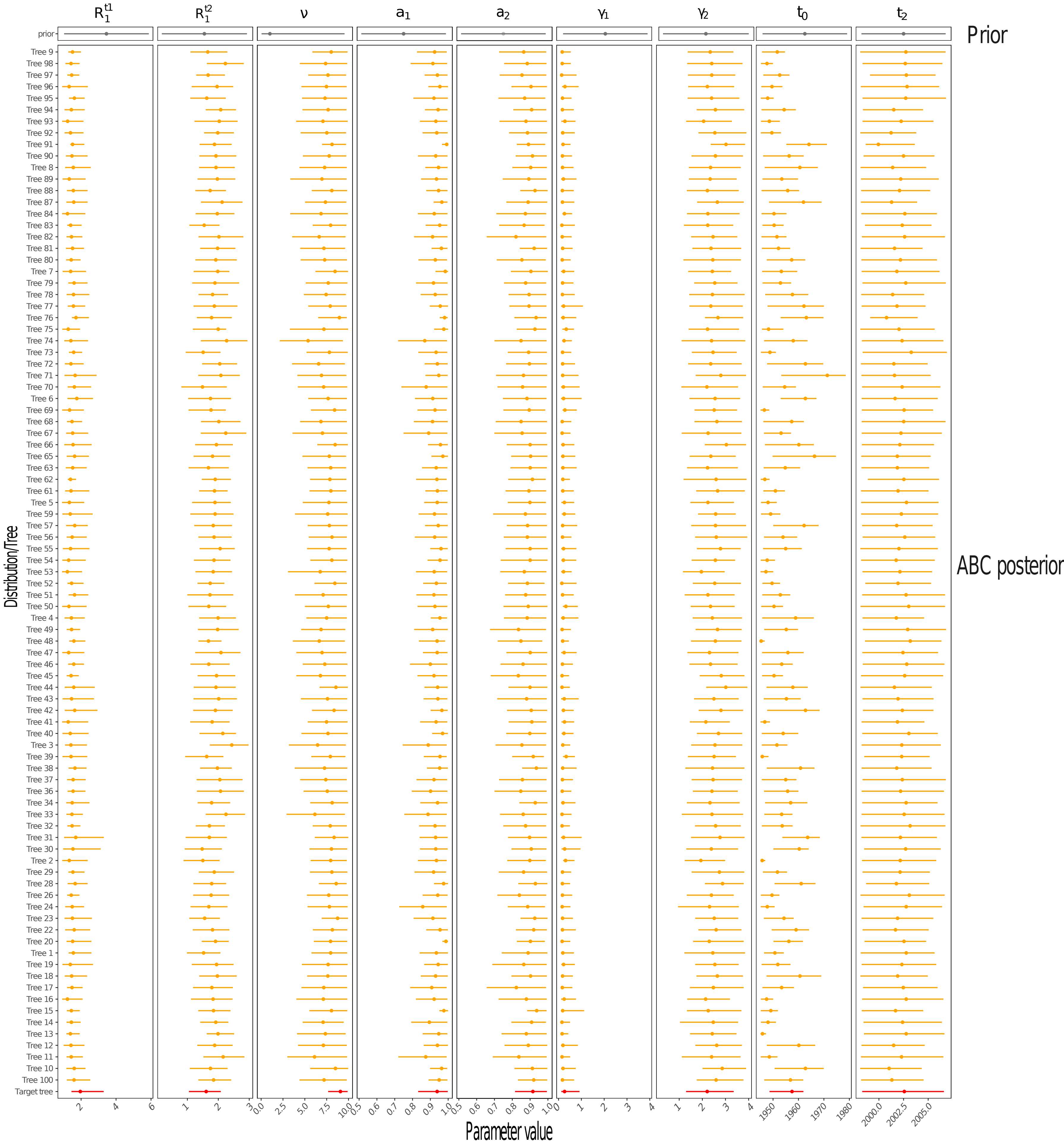

Supplement: S7 Fig — The dots represent the median and the horizontal lines represent the 95% highest posterior density (HPD) of each distribution. Grey distributions correspond to the prior, orange distributions correspond to the different posterior distributions computed from 100 phylogenies drawn at random in the posterior distribution of trees inferred by Beast2 and red distributions correspond to the ABC-EN posterior distributions. (PDF) [file ppat.1009916.s007.pdf]

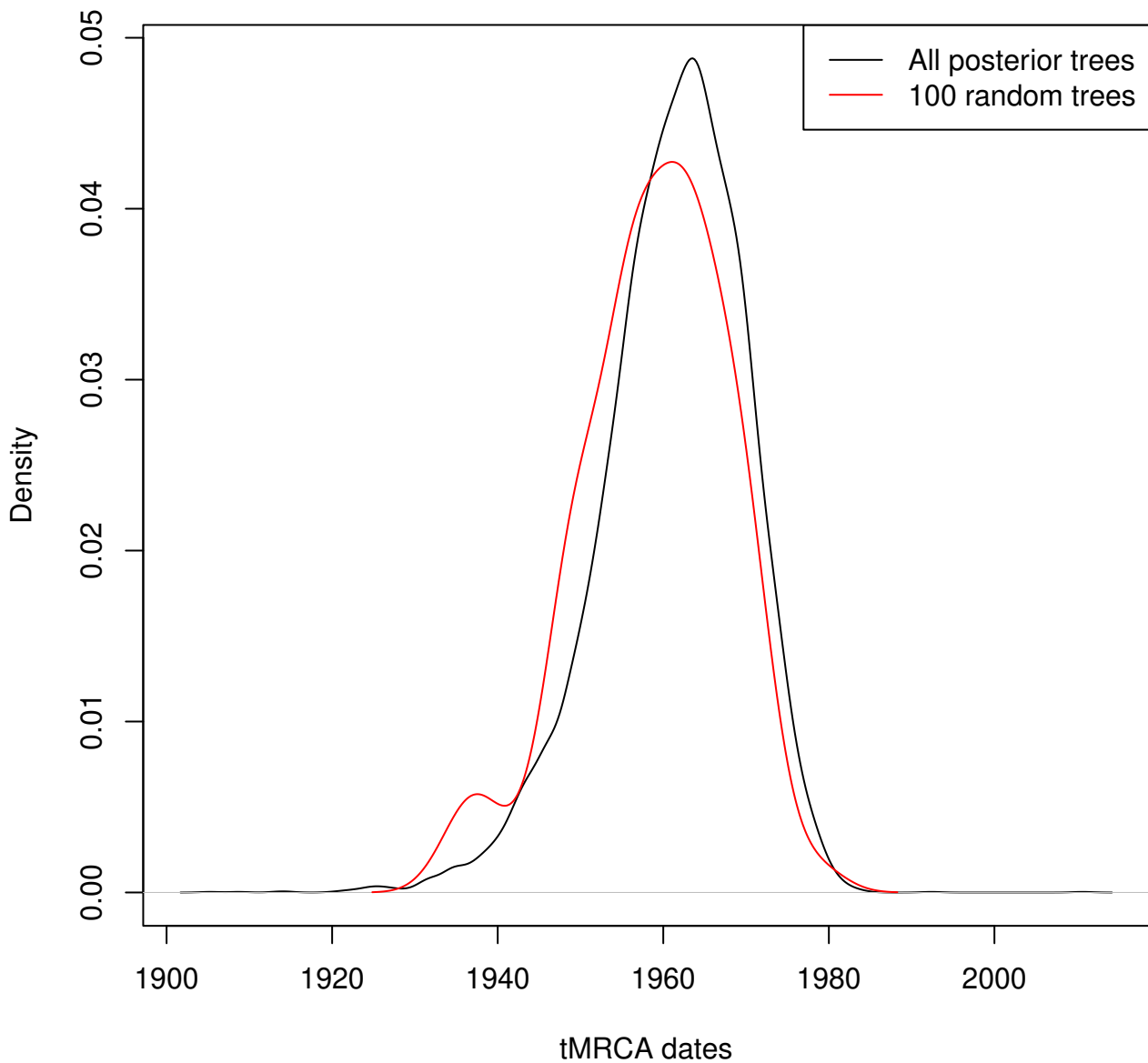

Supplement: S8 Fig — (PDF) [file ppat.1009916.s008.pdf]

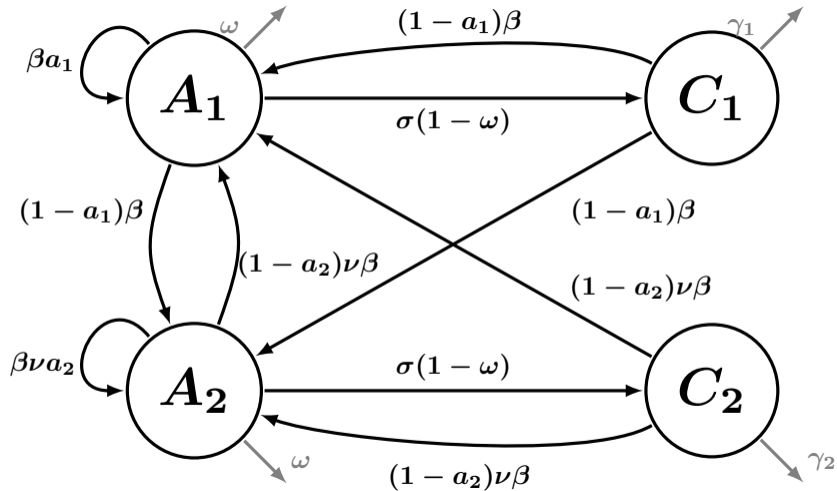

Supplement: S9 Fig — ω is the proportion of infections that clear before becoming chronic, σ is the rate at which acute infections become chronic, and other parameters are identical to those in the main text. The equations governing the dynamics of the system can be written as dAidt=aiβi(Ai+Ci)+(1−aj)βj(Aj+Cj)−σAi and dCidt=σ(1−ω)Ai−γiCi with i ≠ j, β1 = β and β2 = νβ. (PDF) [file ppat.1009916.s009.pdf]

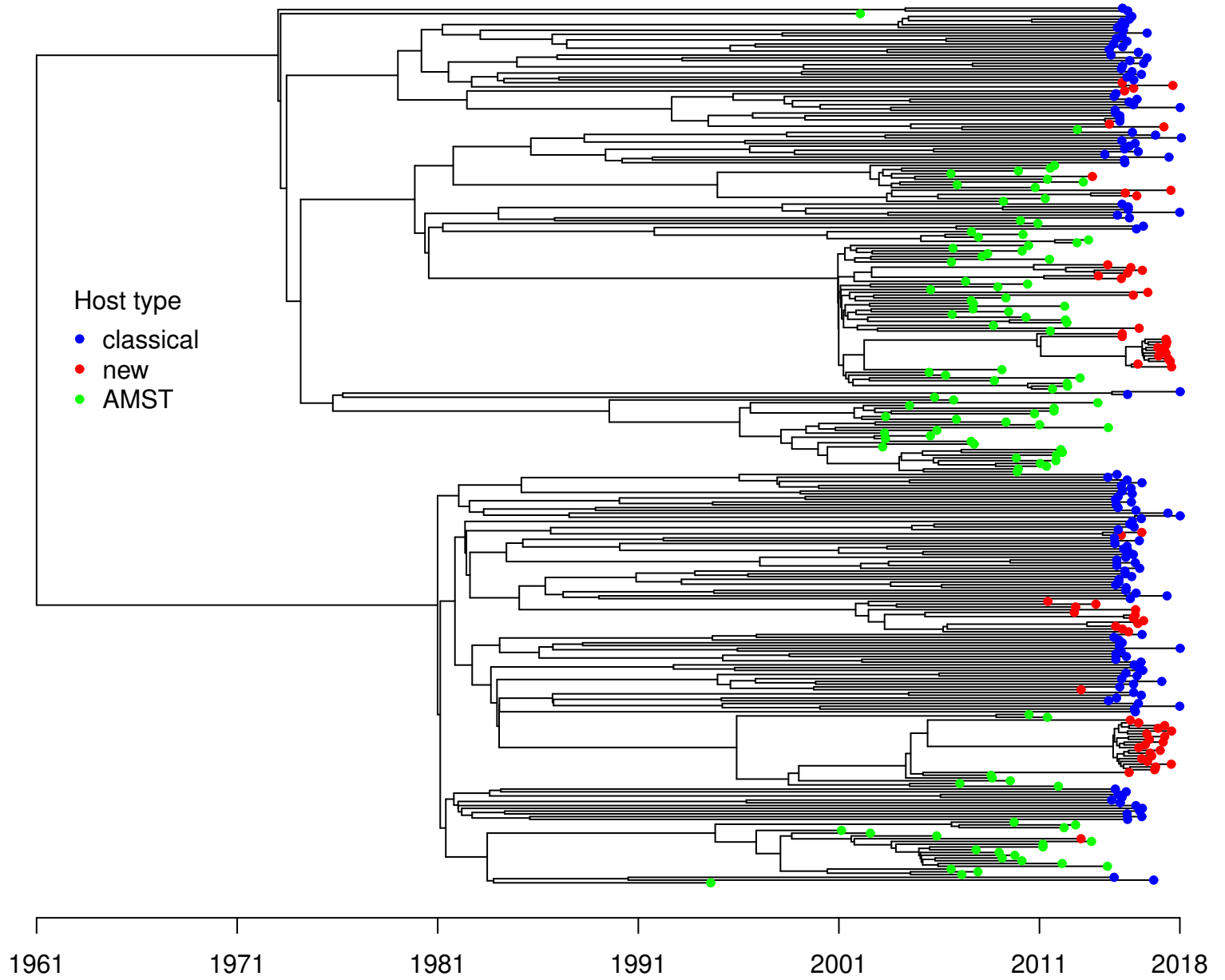

Supplement: S10 Fig — Non-MSM hosts from Lyon are in blue and MSM hosts from Lyon are in red. MSM hosts’ sequences from Amsterdam are in green. Sampling events correspond to the end of black branches. The phylogeny was estimated using Bayesian inference (Beast2). (PDF) [file ppat.1009916.s010.pdf]
