# Supplementary material for: Quantifying transmission dynamics of acute hepatitis C virus infections in a heterogeneous population using sequence data
Source: PLoS Pathog. 2021 Sep 14;17(9):e1009916. doi: 10.1371/journal.ppat.1009916 (PMC8462723; doi:10.1371/journal.ppat.1009916)
Supplement: S1 Table — The parameters R1t1, R1t2 and R1t3 are the reproduction numbers for the non-MSM hosts during the first, second and last temporal intervals respectively. The parameter R2t3 is the reproduction number for the MSM hosts epidemic. γ1 and γ2 are the end of infectiousness rates of non-MSM and MSM hosts respectively. t2 corresponds to the date of the emergence of the MSM hosts epidemic. (PDF) [file ppat.1009916.s011.pdf]

**Table S1. Median values and 95% confidence interval of the posterior distributions of the inferred parameters using the bdmm BEAST2 package.** The parameters $R_1^{t1}$, $R_1^{t2}$ and $R_1^{t3}$ are the reproduction numbers for the non-MSM hosts during the first, second and last temporal intervals respectively. The parameter $R_2^{t3}$ is the reproduction number for the MSM hosts epidemic. The parameters given by $1/(\gamma_1 + \epsilon_1)$ and $1/(\gamma_2 + \epsilon_2)$ corresponf to the effective duration of infectious period of non-MSM and MSM hosts respectively, where $\gamma_1$ and $\gamma_2$ are the end of infectiousness rates and $\epsilon_1$ and $\epsilon_2$ are the sampling proportions of non-MSM and MSM hosts. $t_2$ corresponds to the date of the emergence of the MSM hosts epidemic.

| | $R_1^{t1}$ | $R_1^{t2}$ | $R_1^{t3}$ | $R_2^{t3}$ | $1/(\gamma_1 + \epsilon_1)$ | $1/(\gamma_2 + \epsilon_2)$ | $t_2$ |
|---|---|---|---|---|---|---|---|
| median | 1.54 | 1.51 | 0.68 | 1.05 | 2.0 | 1.50 | 2001.91 |
| 95% CI | [1.37; 1.77] | [1.11; 2.55] | [0.20; 0.94] | [0.67; 1.67] | [1.45; 2.54] | [1.23; 1.81] | [1998.38; 2006.00] |
