# Supplementary material for: Quantifying transmission dynamics of acute hepatitis C virus infections in a heterogeneous population using sequence data
Source: PLoS Pathog. 2021 Sep 14;17(9):e1009916. doi: 10.1371/journal.ppat.1009916 (PMC8462723; doi:10.1371/journal.ppat.1009916)
Supplement: S2 Table — (PDF) [file ppat.1009916.s012.pdf]

Table S2. Table presenting the mean, median values and 95% confidence interval of the inferred posterior distributions of parameters (a) and computed posterior distributions of parameters of the model (b) using a phylogeny with MSM hosts sequences corresponding to the two main clades being removed.

	γ_1	γ_2	a_1	a_2	ν	R_1^{t1}	R_1^{t2}
median	0.24	1.90	0.84	0.82	7.33	2.25	1.58
mean	0.28	2.00	0.83	0.82	7.21	2.35	1.59
95% CI	[0.14; 0.61]	[0.68; 3.67]	[0.63; 0.99]	[0.64; 0.99]	[3.88; 9.88]	[1.73; 3.52]	[1.03; 2.32]

(a) Mean, median values and 95% confidence interval of inferred posterior distributions of parameters of the model using the ABC approach.

	R_2^{t3}	$t_D^{(1),t1}$	$t_D^{(1),t2}$	$t_D^{(2),t3}$
median	1.44	2.09	5.15	0.86
mean	1.59	2.21	13.64	1.70
95% CI	[1.03; 3.06]	[1.13; 3.84]	[2.26; 67.28]	[0.29; 8.74]

(b) Mean, median values and 95% confidence interval of posterior distributions of parameters computed from the inferred posterior distributions
