# Supplementary material for: Quantifying transmission dynamics of acute hepatitis C virus infections in a heterogeneous population using sequence data
Source: PLoS Pathog. 2021 Sep 14;17(9):e1009916. doi: 10.1371/journal.ppat.1009916 (PMC8462723; doi:10.1371/journal.ppat.1009916)
Supplement: S3 Table — In this analysis, the reproduction number of the non-MSM hosts in the most recent time period (R1t3) is also inferred. (PDF) [file ppat.1009916.s013.pdf]

Table S3. Table presenting the mean, median values and 95% confidence interval of the inferred posterior distributions of parameters (a) and computed posterior distributions of parameters of the model (b). In this analysis, the reproduction number of the non-MSM hosts in the most recent time period (R_1^{t3}) is also inferred. The parameters $t_D^{(1),t1}$, $t_D^{(1),t2}$ and $t_D^{(1),t3}$ are the doubling times for the non-MSM hosts epidemic before 1997, after 1997 and after the emergence of the MSM hosts epidemic.

	γ_1	γ_2	a_1	a_2	ν	R_1^{t1}	R_1^{t2}	R_1^{t3}
median	0.27	2.64	0.88	0.81	7.82	1.91	1.76	1.56
mean	0.29	2.68	0.88	0.82	7.57	2.02	1.87	1.56
95% CI	[0.14; 0.51]	[1.34; 3.88]	[0.75; 0.97]	[0.67; 0.99]	[5.23; 9.77]	[1.48; 2.88]	[1.05; 2.86]	[1.01; 2.02]

(a) Inferred posterior distributions.

	R_2^{t3}	$t_D^{(1),t1}$	$t_D^{(1),t2}$	$t_D^{(1),t3}$	$t_D^{(2),t3}$
median	1.15	2.70	3.30	4.488	1.74
mean	1.20	2.90	10.49	24.99	4.60
95% CI	[1.01; 1.64]	[1.56; 4.65]	[1.23; 56.37]	[2.1; 40.03]	[0.47; 26.96]

(b) Computed posterior distributions.
