# Supplementary material for: Quantifying transmission dynamics of acute hepatitis C virus infections in a heterogeneous population using sequence data
Source: PLoS Pathog. 2021 Sep 14;17(9):e1009916. doi: 10.1371/journal.ppat.1009916 (PMC8462723; doi:10.1371/journal.ppat.1009916)
Supplement: S1 Appendix — (PDF) [file ppat.1009916.s014.pdf]

S10 Appendix

GenBank accession numbers of sequences used to infer the viral phylogeny. Each Accession number is associated to a host type ('classical' or 'new') and a sampling date.

Accession number	Host type	Sampling date
KY928360	classical	31/07/2014
KY928361	classical	24/09/2014
KY928362	classical	06/10/2014
KY928363	classical	17/10/2014
KY928364	classical	20/11/2014
KY928365	classical	01/12/2014
KY928366	classical	05/01/2015
KY928367	classical	13/01/2015
KY928368	classical	16/01/2015
KY928369	classical	16/01/2015
KY928370	classical	20/01/2015
KY928371	classical	30/01/2015
KY928372	classical	04/02/2015
KY928373	classical	06/02/2015
KY928374	classical	10/02/2015
KY928375	classical	11/02/2015
KY928376	classical	13/02/2015
KY928377	classical	17/02/2015
KY928378	classical	17/02/2015
KY928379	classical	18/02/2015
KY928380	classical	23/02/2015
KY928381	classical	25/02/2015
KY928382	classical	02/03/2015
KY928383	classical	04/03/2015
KY928384	classical	05/03/2015
KY928385	classical	09/03/2015
KY928386	classical	16/03/2015
KY928387	classical	18/03/2015
KY928388	classical	27/03/2015
KY928389	classical	30/03/2015
MH885712	classical	31/03/2015
MH885713	classical	31/03/2015
MH885714	classical	10/04/2015
MH885715	classical	14/04/2015

Accession number	Host type	Sampling date
MH885716	classical	15/04/2015
MH885717	classical	21/04/2015
MH885718	classical	28/04/2015
MH885719	classical	30/04/2015
MH885720	classical	30/04/2015
MH885721	classical	30/04/2015
MH885722	classical	07/05/2015
MH885723	classical	20/05/2015
MH885724	classical	26/05/2015
MH885725	classical	28/05/2015
MH885726	classical	03/06/2015
MH885727	classical	05/06/2015
MH885728	classical	08/06/2015
MH885729	classical	09/06/2015
MH885730	classical	10/06/2015
MH885731	classical	11/06/2015
MH885732	classical	12/06/2015
MH885733	classical	12/06/2015
MH885734	classical	17/06/2015
MH885735	classical	18/06/2015
MH885736	classical	19/06/2015
MH885737	classical	03/07/2015
MH885738	classical	08/07/2015
MH885739	classical	22/07/2015
MH885740	classical	24/07/2015
MH885741	classical	28/07/2015
MH885742	classical	29/07/2015
MH885743	classical	30/07/2015
MH885744	classical	12/08/2015
MH885745	classical	17/08/2015
MH885746	classical	17/08/2015
MH885747	classical	18/08/2015
MH885748	classical	21/08/2015
MH885749	classical	24/08/2015
MH885750	classical	27/08/2015
MH885751	classical	07/09/2015
MH885752	classical	08/09/2015
MH885753	classical	08/09/2015
MH885754	classical	09/09/2015
MH885755	classical	10/09/2015

Accession number	Host type	Sampling date
MH885756	classical	16/09/2015
MH885757	classical	17/09/2015
MH885758	classical	22/09/2015
MH885759	classical	29/09/2015
MH885760	classical	29/09/2015
MH885761	classical	06/10/2015
MH885762	classical	12/10/2015
MH885763	classical	19/10/2015
MH885764	classical	20/10/2015
MH885765	classical	21/10/2015
MT108308	classical	28/10/2015
MT108309	classical	28/10/2015
MT108310	classical	06/11/2015
MT108311	classical	20/11/2015
MT108312	classical	25/11/2015
MT108313	classical	02/12/2015
MT108314	classical	04/12/2015
MT108315	classical	07/12/2015
MT108316	classical	11/12/2015
MT108317	classical	11/12/2015
MT108318	classical	14/12/2015
MT108319	classical	30/12/2015
MT108320	classical	04/01/2016
MT108321	classical	05/01/2016
MT108322	classical	05/01/2016
MT108323	classical	08/01/2016
MT108324	classical	15/01/2016
MT108325	classical	15/01/2016
MT108326	classical	19/01/2016
MT108327	classical	03/02/2016
MT108328	classical	05/02/2016
MT108329	classical	05/02/2016
MT108330	classical	16/02/2016
MT108331	classical	16/02/2016
MT108332	classical	19/02/2016
MT108333	classical	26/02/2016
MT108334	classical	07/03/2016
MT108335	classical	07/03/2016
MT108336	classical	30/03/2016
MT108337	classical	01/04/2016

**Accession number**	**Host type**	**Sampling date**
MT108338	classical	01/04/2016
MT108339	classical	07/04/2016
MT108340	classical	18/04/2016
MT108341	classical	25/04/2016
MT108342	classical	26/04/2016
MT108343	classical	19/05/2016
MT108344	classical	25/05/2016
MT108345	classical	26/05/2016
MT108346	classical	27/05/2016
MT108347	classical	03/06/2016
MT108348	classical	06/06/2016
MT108349	classical	07/06/2016
MT108350	classical	13/06/2016
MT108351	classical	21/06/2016
MT108352	classical	01/07/2016
MT108353	classical	04/07/2016
MT108354	classical	07/09/2016
MT108355	classical	08/09/2016
MT108356	classical	06/01/2017
MT108357	classical	08/02/2017
MT108358	classical	01/06/2017
MT108359	classical	05/09/2017
MT108360	classical	22/09/2017
MT108361	classical	10/10/2017
MT108362	classical	24/04/2018
MT108363	classical	24/04/2018
MT108364	classical	02/05/2018
MT108365	classical	02/05/2018
MT108366	classical	02/05/2018
MT108367	classical	03/05/2018
MT108368	classical	24/05/2018
MH885654	new	27/07/2011
MH885655	new	21/01/2013
KY928329	new	15/02/2013
KY928344	new	23/05/2013
KY928348	new	24/05/2013
KY928322	new	16/12/2013
MH885656	new	18/02/2014
MH885657	new	04/04/2014
KY928330	new	22/09/2014

Accession number	Host type	Sampling date
MH885658	new	22/10/2014
KY928355	new	13/02/2015
MH885659	new	26/05/2015
KY928356	new	27/05/2015
MH885660	new	10/06/2015
KY928352	new	12/06/2015
MH885661	new	12/06/2015
MH885662	new	15/06/2015
KY928313	new	24/07/2015
KY928314	new	06/08/2015
MH885663	new	16/09/2015
MH885664	new	05/10/2015
KY928336	new	14/10/2015
KY928338	new	05/11/2015
KY928350	new	16/11/2015
MH885665	new	29/12/2015
MH885666	new	30/12/2015
MH885667	new	05/01/2016
MH885668	new	03/02/2016
MH885669	new	10/02/2016
MH885670	new	03/03/2016
KY928357	new	23/03/2016
KY928341	new	24/03/2016
MH885671	new	06/04/2016
KY928354	new	07/04/2016
KY928311	new	15/04/2016
KY928335	new	31/05/2016
KY928346	new	02/06/2016
MH885672	new	13/06/2016
MH885673	new	05/07/2016
KY928349	new	03/08/2016
KY928331	new	01/09/2016
MH885674	new	14/09/2016
KY928324	new	21/09/2016
KY928353	new	19/10/2016
MH885675	new	04/11/2016
KY928351	new	30/11/2016
KY928343	new	23/01/2017
MH885676	new	10/02/2017
KY928325	new	22/03/2017

Accession number	Host type	Sampling date
MH885677	new	27/03/2017
KY928333	new	05/04/2017
KY928345	new	03/05/2017
KY928315	new	27/06/2017
MH885693	new	10/07/2017
MH885695	new	19/07/2017
MH885697	new	24/07/2017
MH885698	new	25/07/2017
MH885699	new	16/08/2017
MH885700	new	17/08/2017
MH885701	new	21/08/2017
MH885702	new	30/08/2017
MH885703	new	27/07/2017
MH885704	new	03/11/2017
MH885705	new	14/11/2017
MH885707	new	27/11/2017
MH885711	new	20/12/2017
MT108306	new	22/11/2017
MT108307	new	27/11/2017
