# Supplementary material for: Quantifying transmission dynamics of acute hepatitis C virus infections in a heterogeneous population using sequence data
Source: PLoS Pathog. 2021 Sep 14;17(9):e1009916. doi: 10.1371/journal.ppat.1009916 (PMC8462723; doi:10.1371/journal.ppat.1009916)
Supplement: Attachment — Submitted filename: PLoSPath_responses.pdf [file ppat.1009916.s015.pdf]

Reviewer's Responses to Questions

Part I - Summary

Reviewer #1: This is a very interesting paper that describes a novel phylodynamic approach. The authors do not explicitly combine an evolutionary and epidemiological model into one approach, in the manner of more popular coalescent and birth-death phylodynamic models (e.g. the work of Erik Volz, Tanja Stadler, David Rasmussen, etc.). Rather, the authors use Approximate Bayesian Computation to evaluate sets of epidemiological parameters using a given phylogeny as the "target" phylogeny (the empirical, known, phylogeny is considered fixed before the ABC is started). This really is quite a fascinating study, and while I do not have the full expertise to comment exhaustively on all of the statistical approaches included, I believe it will be an important addition to the literature. It is true that genomic epidemiology has hesitated to use phylogenies as calibration tools (Art Poon's work with HIV notwithstanding, and Emma Saulnier et al's work from 2017), but this might be an important tool in the near future.

Thank you for the positive appreciation!

Reviewer #2: In this manuscript, Danesh et al. apply Approximate Bayesian Computation to time collaborated Hepatitis-C (HCV) phylogenies to estimate key epidemiological parameters of the recent HCV epidemic in Lyon, France. HCV is currently spreading in a heterologous population of "classical" hosts (i.e. those infected as the result of intravenous drug use or blood transfusion) and "new" hosts, typically MSM. Viral phylogenetics disentangles these co-occurring epidemics and shows that they are driven by two separate processes in separate populations. The authors then use ABC to estimate the parameters of a more complex compartmental model than is feasible using classical phylodynamic approaches. The approach is well-suited to the problem of dissecting and characterizing the recent epidemic, and the findings provide important insights into a growing public health concern. The fact that the "new" and "classical" epidemics are distinct events that rarely mix is an important and robust finding.

Thank you for the positive feedback!

However, there are caveats to the specific claims made regarding the parameter values that detract from an otherwise sound study. In particular, the placement of the phylogenetic tree in the context of global HCV circulation and the impact of the time constraints imposed on the model should be explored.

We inferred a phylogeny using European sequences (from Amsterdam) in addition to ours to assess the structure of the Lyon epidemic. We find that the epidemic we analyse does not appear to be monophyletic, which is consistent with earlier results on the circulation of HCV in Europe. However, the non-MSM (former "classical') hosts tend to be monophyletic.

=> *We added the European phylogeny in the Appendix and mention its structure in the Discussion.*

Reviewer #3: The authors use phylodynamic methods to shed light on the HCV epidemic oin Lyon, France. They identify two host types, classical and new ones, the latter of which appears to have a drastically faster epidemic doubling time, calling for a quick response by public health authorities. This study is important and well within the scope of this journal.

Thanks!

Part II – Major Issues: Key Experiments Required for Acceptance

Reviewer #1: No major issues.

Thanks!

Reviewer #2: 1) The analysis relies on a phylodynamic reconstruction of hosts sampled only in the recent past and assumes a single exponentially growing epidemic in Lyon since the late 1960s. What is known about HCV import into Lyon during the decades covered by the phylogeny?

The reviewer is correct in that our model assumes exponential growth. However, we wish to stress that there were two time periods where the epidemic was assumed to grow (or decrease) exponentially (before and after 1997). In the revised version, we also added an analysis with 3 time periods (before 1997, between 1997 and 2002, and after 2002).

Overall, little is known about import into Lyon. The European phylogeny mentioned above does show that these imports exist (at least between Lyon and Amsterdam). Another indication that major epidemic trends have taken place is that there has been a replacement of circulating HCV genotypes. Indeed, a study (doi:10.1111/j.1365-2893.2005.00605.x) on a large French population with chronic hepatitis C between 2000 and 2001, showed a large decrease in frequency of genotype 1b (the most common one at the time) and an increase of genotypes 1a and 4, with genotype 1a being mostly associated to Injecting Drug Users (IDU). At that time, the authors observed for the first time that the most predominant reported mode of contamination in France was IDU. We also know that immigration contributed to the diffusion of new genotypes. For example, some variations were observed with a higher proportion of genotypes 2 and 4 in regions in France with more immigration and these genotypes were mostly found in patients of Central African origin.

Overall, our phylogenetic result with the Amsterdam sequences suggests that there is room for a phylogeography study to better understand the structure and history of French HCV epidemics.

=> We now mention in the Discussion the limitations regarding the assumption of exponential growth or decay, as well as the unknown regarding the import of HCV in Lyon and its links with the phylogeography of this virus in Europe.

Does the early growth indicated by the phylogeny correspond to endemic growth in Lyon, or is it the result of a combination of endemic growth and foreign epidemics that were imported into Lyon?

Based on the fact that our sequences do not represent a monophyletic clade in the European HCV epidemic, this may indeed reflect multiple importations and the circulation of the virus at a wider scale at the time.

=> We now mention the potential migration events in the Discussion.

Earlier samples and samples from different geographic regions should be included to verify the dataset represents a single epidemic in Lyon that was seeded in the 1960s. (These context-samples could be pruned out before fitting the ABC model).

As indicated above, we constructed a European phylogeny. For the ancestral sequences, we indeed do not have a single origin. However, for the more recent sequences, we do have monophyletic clusters.

From a technical standpoint, an alternative could be to analyse the recent clusters separately (as, for instance Kuenhert et al 2018 did to quantify the fitness cost of HIV mutations) but this would also be decreasing the level of information, which is why we prefer to analyse all sequences jointly.

=> We now discuss this in the text by saying that the early growth most likely captures HCV spread at a wider level, but that recent sequences correspond to local epidemics.

Related to the point above, the recent, “new” host epidemic appears to be driven by two heavily sampled clades, which have a large number of branching events in the very recent past. The authors account for differences in sampling proportion between demes; however, they do not account for biased sampling within demes. If these clades are known or probable epidemiological clusters then including them in the analysis would lead to an artificial increase in the growth rate of the “new” epidemic. Is anything known about these clusters? It would be interesting to quantify the impact these clades have on the growth estimates by removing them from the tree and estimating their growth separately.

Unfortunately, the level of detail is not sufficient to focus on specific events, i.e. the aforementioned clades.
The variation of the sampling rate between clades is indeed a very timely research question. For instance, if these correspond to different screening facilities that use different methods (e.g. backwards contact-tracing, forward contact-tracing, random sampling), clearly the resulting phylogeny will differ. Here, the sampling was performed by the same public health institutions, which is why we assume it to be homogeneous, at least for the MSM (former

“new”) epidemic. We know that HIV patients or those undergoing PREP are mostly patients who are already being followed.

=> *We better describe how the sampling is performed for the new epidemic.*

We also reran the whole analysis without the two clades, which meant removing 13.6% of the data and 57% of the MSMS (former “new”) epidemic data. The results we obtained show the same quantitative tendencies with the same differences between the duration of the effective infectious period for the new and the classical hosts, a more rapid spread of the new epidemic and a high level of assortativity.

=> *We now mention this analysis and the results in the Discussion.*

2) The authors compare the doubling time of the “new” epidemic with that of the “classical” epidemic after the advent of rapid tests in 1997. There are several issues with this comparison. The method used here assumes an exponentially growing epidemic; however, the “classical” epidemic is decades old at this point. The authors should discuss the impact of these assumptions on their estimates.

=> *We now mention that the non-MSM (former “classical”) epidemic is most likely in a different stage given that it emerged before. We also mention that considering three time periods instead of 2 has little effect on the results regarding the non-MSM epidemic trends.*

Also, one of the parameters in this comparison, gamma, is assumed to be constant throughout the epidemic. This seems valid for the “new” epidemic which is young and growing, but not for the “classic” one which spans 60 years and has been impacted by non-pharmaceutical interventions. As the main conclusions stem from a difference in the gamma parameters any bias in these estimates should carefully be considered.

We indeed assumed that the duration of the infectious period (1/gamma) is constant, for a given host type, over the time period considered. The motivation for this assumption was that our inference power remains limited and we preferred to allow the reproduction number to vary. The reviewer is correct in that the gamma for the non-MSM (former “classical”) hosts might have increased over the years with the implementation of non-pharmaceutical interventions. Therefore, the infection duration value we present is an upper bound and it could be lower in the more recent time period than in the oldest time period.

To test this potential bias, we ran an ABC analysis with the variation of gamma for the non-MSM (former classical) hosts before and after 1997, corresponding to the first and second time periods. The results obtained showed no difference between these parameters. Further data will be needed to investigate this trend in details.

=> *We now mention that the variation in the effective infection duration is limited.*

3) The manuscript reports that the transmissibility of the “new” epidemic (nu) is ~10 times that of the classical epidemic. This estimate appears to be at the limit imposed

by the prior. Is there any reason to limit the prior to a range of 0,10? If not, a more diffuse prior should be used.

The posterior distribution is indeed close to the limit. The motivation for the prior shape, based on suggestions from the PCI reviewing process, was to make sure that we put the same weight in the prior on having a new epidemic growing more slowly than the classic one (nu in [0,1[ ) or faster than the classic one (nu in [1,10]). Here, the key result is that clearly the new epidemic is spreading faster. Given the number of sequences, it is unlikely that we can obtain a more accurate estimate.

=> *We now better explain the motivation for the range and the qualitative importance of the results.*

The estimate also relies on determining the origin of the "new" epidemic (t2) (which the authors state can not be estimated accurately) and compares parameters in this time frame to those driving the "classical" epidemic in other periods. The methods are unclear, are the classical parameters also estimated in the final time range [t2,t3]? If not, why not. The authors should discuss what this origin represents.

The main parameter related to the non-MSM (former "classical") epidemic (R0_1) was estimated before and after 1997. The t2 parameter corresponds to the emergence of the MSM (former "new") epidemic. We do indeed assume that there are no major changes in the classical epidemic related to the emergence of the "new" epidemic (between t2 and t3). Also, we made this assumption to not to have too many parameters to infer, given the number of sequences.

We reran the inference by inferring a third parameter (R0_1^t3) to have the classical and new epidemic estimated on the same time period. The results of this analysis show that the classical epidemic keeps decreasing. We find the same differences that we observed initially between the duration of the effective infectious period for the new and the classical hosts and between the doubling times for the new and classical hosts.

The tree suggests that the "new" epidemic is the result of multiple introductions into the "new" population, not a single origin event. Judging by the tree in Figure 1, some of these introductions could date back to the late 1970s. However, by setting nu to 0 until the early 2000s, the "new" epidemic is fixed at no growth until the recent past. What is the impact of estimating the growth of the "new" epidemic over the entire period shown by the tree?

When focusing on the clusters of MSM (former "new") hosts, the median date of origin of the corresponding monophyletic clades appears to be posterior to 1998. The long introductions seem to be more related to isolated sequences from "new" hosts and could either reflect new introductions from another area, or low sampling associated with the transmission chain. Finally, as indicated in the first version of the epidemic, this hypothesis is supported by field epidemiology studies.

=> *We now further discuss the phylogeny itself and the dating of the clusters associated with the new epidemic.*

Reviewer #3: 1) a) Please rename "new" and "classic" hosts - otherwise this will be outdated soon.

Initially the epidemics were labelled as men having sex with men (MSM) and injecting drug users (IDU) in reference to the majority risk group in each epidemic. Although clearer and less likely to become outdated, this also simplifies the picture very much.

=> *To accommodate both aspects, we now refer to the first epidemic dominated by transmission in IDU risk-groups as the epidemic in the non-MSM host type and the second epidemic dominated by MSM transmission as the epidemic in the MSM host type.*

b) The group assignment seems difficult, since "chemsex" has recently been associated with injecting drug use as well. Please discuss this more thoroughly.

The host type assignment is based on different factors and rather than the route of transmission, it mostly relies on the epidemiological profile. The term “chemsex” is associated with drug use in the sexual context, either via intravenous (IV) (= "slam"), or nasal, or other routes (rectal, oral). There is a direct risk of HCV transmission through IV or nasal use (contamination of equipment), and possibly rectal. But in any case, one cannot differentiate the respective part of drug use in this sexual context from the risks represented by "hard" sexual practices, group sex or "simple" anal sex, which as such is recognized as a risk factor. This is why we refer to "sexual" transmission of HCV, because it is considered that it is probably a blood transmission, occurring during sexual intercourse, with overlapping risk factors (group sex + anal sex +/ BDSM +/- drugs…)

=> *We now further discuss the limitation of assigning a specific risk group to one epidemic, using chemsex as an example.*

2) Could the estimated difference in doubling times be biased by the fact that the "new" host epidemic started very recently? Please test this with simulations in which "classical" hosts have a short doubling time and "new hosts" have a long doubling time (but with a higher sampling rate in new hosts than classical ones, as the authors say that the "sampling rate of ’new’ hosts may be higher than that of 144 ’classical’ hosts" l.144)

Biologically, we see that the second epidemic bursting in a short amount of time. Therefore, it seems logical that it has a lower doubling time.

To further explore this and address the reviewer’s concern, we simulated different phylogenies using the real sampling dates and varying the doubling time differential (with different longer doubling times for new/MSM hosts than classical/non-MSM hosts). We then performed our ABC analysis for each “target” simulated phylogeny.

We see in the figure below that the higher the doubling time of new/MSM hosts is compared to that of the non-MSM/classical hosts, the less the ABC approach retains simulated data. If

few simulations are kept, it means that the prior values for the parameter do not allow to simulate the target dataset. This is obviously an issue because for the regression part of the ABC to run, we do need enough simulations. Strikingly, to keep a significant number of simulated data we need a lower doubling time for the MSM/new hosts. This further illustrates that there is a constraint in the data structure.

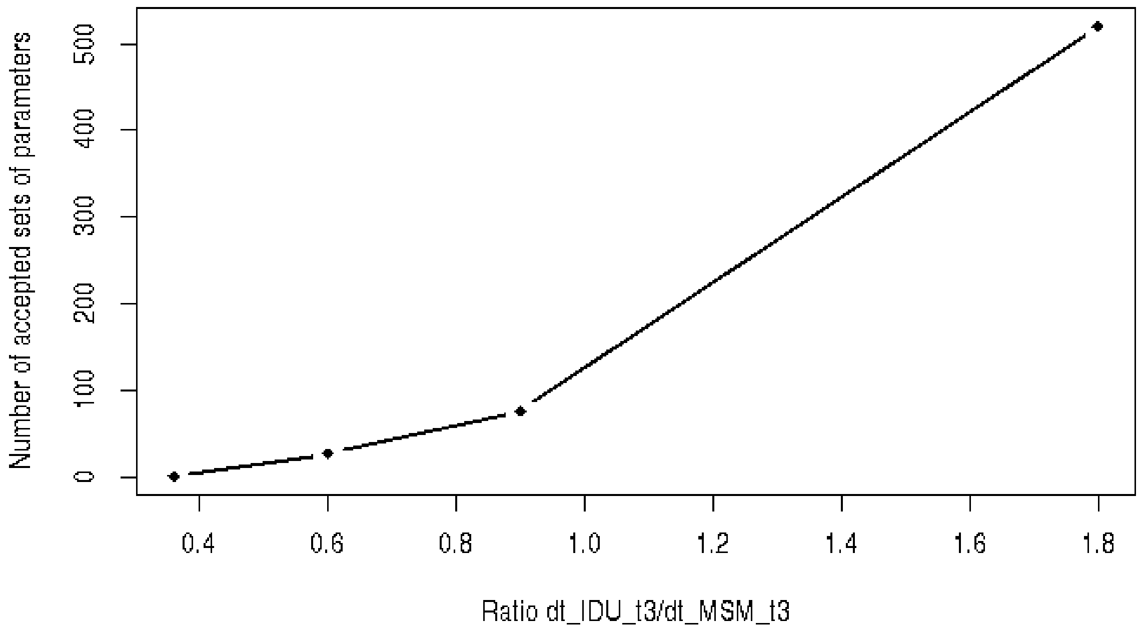

3) (related to 1 & 2) The authors state that the type assignment was mainly done by infection stage (acute vs chronic) - please perform a sensitivity test in which the acute infections in the third (most recent) time interval are denoted as "new" hosts, to test if this drives the difference in epidemic doubling time. the sensitivity test the authors did is not enough, because it would also assign older acute infections to the new host type (which does not seem to reflect the assignment in the real data)

This is a valuable suggestion, unfortunately we do not have the exact information regarding the stage at which the individuals were sampled.

However, to address this concern that the stage of the infection could be driving the dynamics, we created an alternative model without host types but with an acute and a chronic infection phase. In other words, we interpret the data by making the simplifying assumption that all new hosts were actually hosts sampled during their acute phase and all classical hosts were hosts sampled in their chronic phase. With such a model, it was impossible to even simulate the data. This makes sense because of the very strong assortativity we see in the phylogeny between new and classical hosts (this is lost with the acute/chronic dichotomy since all hosts go through the acute stage at some point). It is also an illustration of the added value of the phylogenetic information.

=> *We now mention that a model with only infection stages almost completely fails to reproduce the phylogenetic data.*

4) The estimated recovery rates may be biased by the lack of a sampling process in birth-death model? If sampling is much faster in new vs classical hosts, this will lead to the recovery rate being estimated as faster as well, and with that reduce the epidemic doubling time. If the ABC implementation doesn't allow for that, this could be tested in bdmm (with the target tree fixed)

To address this concern, we implemented our model in the BDMM framework in Beast. This was made possible thanks to critical help from Julija Pecerska, Denise Kühnert, and Louis du Plessis (all listed in the acknowledgments). Without their help, it would have been impossible to parameterise the XML input file correctly since this required many manual edits.

=> *We analyse the results of the BDMM inference and compare it to ours.*

5) Do the authors have any data on further co-infections e.g. with syphilis - as this increases the risk of transmission? How common are such co-infections in the study population? Please add to the discussion.

The dataset is currently limited to HIV and HCV. Incorporating additional patient information would almost require another study because it would require obtaining the relevant ethical agreements and going through the individual patient files.

We do not have any data regarding other co-infections.

Part III – Minor Issues: Editorial and Data Presentation Modifications

Reviewer #1: 1. Page 1 in the Background, the authors describe incidence patterns across time and across populations, but don’t state the actual incidence estimates, or case counts. These would be helpful for context.

We now refer to data from Pradat et al published in AIDS that presents some trends regarding HCV incidence in Lyon. The main figure is shown below that indicates a pronounced increase in MSM hosts, especially for first infections

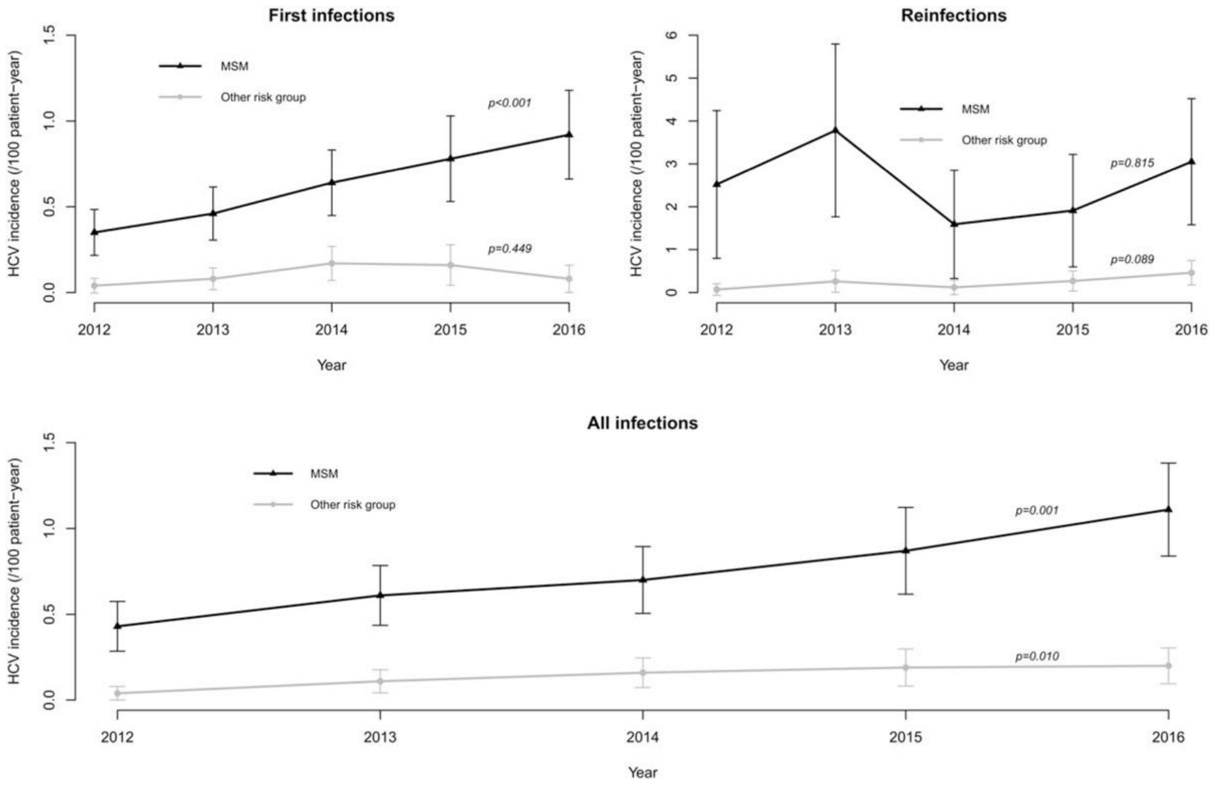

=> *We mention in the Introduction that HCV incidence has been increasing in Lyon in MSMs, especially for first infections.*

2. For Figure 2, in the legend, can you add the definitions of the variables?

We added the variables definitions in the legend.

3. Re: "The phylodynamics approach also allows us to infer the duration of the infectious period for each host type." and "3.85 years [1.09;8.33] for 'classical' hosts (parameter 1/γ1) and 0.45 years [0.30;0.77] for 'new' hosts."
The results for the duration of the infectious period seem odd, if not in results then in simple definition. I would have thought that the duration of the infectious period is generally a static parameter, similar across populations and time, that has more to do with intrahost virological and immunological processes than with epidemiological or network processes. But the authors' inference of such large differences in the infectious period between the 'classical' and 'new' hosts implies that something virological is underlying this pattern. Maybe I am simply misunderstanding their model and terminology, but this seems to need a bit more discussion. The SIR model includes this parameter as γ1 and γ2, and defines it as the rate of removal from the infectious compartment (I1 or I2) via infection clearance, host death, or change in host behaviour (e.g. condom use). This definition makes sense, and is consistent with standard SIR models. So maybe I am just quibbling about the terminology.

Our formulation of “infectious period” was indeed unclear. As pointed out in one of the previous comments, these estimates include non-pharmaceutical interventions (e.g. promoting condom use) that de facto reduce the average time during which an infected person transmits the infection. Therefore, this duration refers more to the generation time, (i.e. if there is a transmission event, when does it occur during the infection?). However, there is also a difference between the two because the host sampling will also contribute to the generation time (we assume that sampled hosts do not transmit anymore). Overall, formally, 1/gamma can be interpreted as the generation time if the effect of sampling is neglected.

=> We now refer to gamma as the “effective” infectious period to clarify that it differs from the biological infectious period.

4. I read with interest the description of the authors' attempt to use BEAST2 (and jointly estimate the phylogeny and epidemiological parameters) for their dataset. And the sentence, "We were unable to conclude anything from 215 this analysis." is really something. At the risk of making the paper longer, it would be informative to hear what aspects of the HCV data that the authors thought was at fault for the inability of these methods to help. Particularly the structured coalescent approach.

Thanks to the help of three young researchers, we were finally able to parameterise the XML input file of Beast in order to estimate parameters for a relevant model. We agree that this does point to the limitations of Beast for widespread usage, especially because of the required detailed tweaking of the XML file, but we decided not to discuss it in detail.

Reviewer #2: Abstract: “new” and “classical” hosts should be defined before being used.

=> As requested by Reviewer #3, we clarified the definition of the two host types and renamed them to improve clarity.

It would be very useful to show the inferred trajectories of the number of cases in both populations overtime. As the authors state, these metrics are difficult to interpret in a heterogeneous population without phylogenetic approaches. It would be nice to see how the presented analysis augments classical approaches.

=> We used the posterior distribution to generate trajectories of host types over time. We show on the same plot the inference made using Beast2 and the BDMM model.

It would be nice to include a table of the key parameter estimates (gamma, beta, nu, a) and their statistics (R0 and tD) for each population and period.

=> We added a table similar to Table 1 showing the median and 95% HPC intervals for all the parameters.

Related to (3) above, what are the initial, infected population, sizes used in the model, and do they account for the multiple introductions into the "new" host population before the estimated origin of that epidemic (t2)?

The epidemic is assumed to start from a single infected individual at t0. Regarding the second type of hosts (new/MSM), there is no initial size because they continuously emerge from the first (classical/IDU) host type. Indeed, since the host types refer to host behaviour and not to virus properties, we can easily assume that the epidemics in the first and second host types are linked.

=> *This initial size issue is now clarified in the manuscript.*

The tree in Figure 1 forms the basis of the study and is generated from ~400 basepair region of the genome. There is likely a large amount of uncertainty in the topology and node heights. Posterior probabilities and / node heigh bars should be shown in some way to indicate uncertainly in the data.

=> *Posterior probabilities are now indicated in the phylogeny.*

Supplemental Figure 3: It would be nice to see the beta parameters directly included in the correlation matrix.

Beta is not estimated directly but we can obtain a distribution from R0 and gamma.

=> *We now show beta in the correlation matrix.*

The set of 100 sampled trees is a nice addition. Is it feasible to estimate the one distribution of parameter values across all these trees as opposed to estimating the parameters for each one separately?

We can estimate a distribution from a target phylogeny only, not from a set of target phylogenies.

Supplemental Figure 8: Is there any explanation for why the distribution of the tMCRA dates of the 100 randomly sampled trees does not match that of the posterior, other than unlucky sampling?

Our interpretation is that the variation in the tMRCA reflects the uncertainty in the posterior distribution from Beast.

Line 174) "new" should be "classical"

Yes thank you.

Figure 2 legend: The thinner the posterior distributions represent more precise estimates but not necessarily more accurate ones.

=> *Yes, this is corrected in the manuscript.*

Reviewer #3: - Please clarify if all 'classical' hosts from Lyon, too?

=> *Yes, all the non-MSM/classical hosts were sampled in Lyon. This is clarified in the text.*

- l.258 "strict clock rate fixed at 1.3 · 10−3 based on data from Ref. [44] "

=> *We added the unit for the clock rate.*

- l 279: doubling time estimation assumes we're still in exponential phase, which is true for new hosts but not for classical ones - please add as caveat to discussion

=> *As explained above, we now specify the limitations about the exponential growth assumption.*

- Table 1: Please add which prior is used on t2.

Our apologies for failing to mention this: t2 is chosen in a uniform prior between t1 (1997) and t3.

=> *The prior is now indicated in the text.*

- How were the priors for R1 chosen (interval 1: Unif(0.9, 6); interval 2: Unif(0.1, 3))?

The uniform shape of the priori was chosen to minimise the initial level of information.

The boundaries were adjusted after preliminary runs to avoid performing useless simulations (i.e. have too wide intervals) or truncating the posterior distributions (have too small intervals).

Notice that the simulation step was already informative on the biological relevance of extreme parameter values. This is also clear from our new discussion revolving around the number of parameter sets kept in the ABC approach.

- S9_Fig is missing arrows from C1>A1 and C2>A1 indicating transmission from chronic to acute individuals within the same host compartment

=> *Thank you for noticing this! We made the correction.*

- ll. 119-120 "if the epidemic spreads rapidly in ‘classical’ hosts, it requires a slower spread in ‘new’ hosts to explain the phylogeny" - and vice versa (which seems to be the case here)

=> *We reformulated and added a clear reference to the associated parameter effects to improve clarity.*

- l. 123: "LONGER doubling time" would be more precise than "lower doubling time"

=> *Yes thank you.*

- ll.188-189: "which is already faster than the classical epidemics" - please clarify what is meant here

=> *We reformulated the sentence to improve its clarity.*

- ll 209ff: Stadler & Bonhoeffer did this already in 2013, doi: 10.1098/rstb.2012.0198 using an ML approach on fixed trees

=> *Yes thank you, this study is now cited in the manuscript.*

- l 215: the problems described here may be overcome by using bdmm on the fixed target tree, which would be good to have for comparison. there is no doubt that the approach presented here is useful, but the authors seem to imply here that there are no good alternatives, which is not true. please rephrase.

=> *We now use the BDMM approach thanks to external help to parameterise the XML file and edited the sentences accordingly.*
